# Does anxiety improve during weight restoration in anorexia nervosa? A systematic review

**DOI:** 10.1186/s40337-015-0046-2

**Published:** 2015-03-18

**Authors:** Sarah Kezelman, Stephen Touyz, Caroline Hunt, Paul Rhodes

**Affiliations:** School of Psychology, University of Sydney, Sydney, Australia

**Keywords:** Eating disorder, Anorexia nervosa, Weight, Anxiety, Nutritional rehabilitation

## Abstract

Weight restoration is considered a principal outcome for treatment of Anorexia Nervosa (AN) due to the significant physiological disturbances resultant from acute states of malnutrition. Treatment outcomes for populations with AN are relatively poor, with increasing evidence suggesting that weight restoration alone is insufficient for long-term recovery. Research aimed at understanding the psychological sequaele of AN, in particular during weight restoration, nevertheless remain scarce. This systematic review aimed to evaluate existing research regarding anxiety symptoms during treatment for AN, and the relationship of anxiety symptomology and weight restoration. Twelve articles were identified from a systematic search of three electronic databases (PsycINFO, MEDLINE, and Web of Science), and were eligible for inclusion. Study methodology, results and quality were reviewed. Results regarding change in anxiety symptomology were inconsistent, though evidence did not support a relationship between anxiety change and weight restoration. Reasons for these inconsistencies and limitations of included studies were reviewed. Further research is warranted to elucidate the role of anxiety in AN and its implications for treatment and longer-term outcome.

## Review

Anorexia Nervosa (AN) is a severe ego-syntonic psychiatric illness [1] characterized by a refusal to maintain a minimally normal body weight and an intense fear of gaining weight or becoming fat (DSM-5) [2]. Severe malnutrition associated with AN results in a number of deleterious physiological outcomes [3,4] and moreover, is associated with AN having the highest mortality rate of any psychiatric disorder [1,5,6], with standardized mortality ratios (i.e., the ratio of observed to expected deaths) estimated at approximately 5.86 to 8.85 [7,8]. Inpatient admissions aimed at correcting the biological sequelae of malnutrition are thus indicated for individuals with AN at imminent risk of severe medical compromise [9,10].

At present treatment outcomes for individuals with AN is relatively poor with longitudinal research demonstrating that high dropout rates [11-13], high rates of re-admission [14,15], and low rates of long-term holistic recovery [5,7] should be considered normative [16]. Furthermore, evidence suggests that individuals who are discharged or dropout from inpatient wards prior to achieving weight normalization are more likely to be re-hospitalised and have poorer treatment outcomes in the long-term [17,18]. Importantly, low body mass index (an indicator for those individuals most in need of nutritional rehabilitation) at referral has been associated with poorer treatment outcome and prognosis [19]. While weight restoration is unquestionably a fundamental clinical aspect of AN that needs to be targeted in treatment interventions, there are increasing suggestions that changes in weight restoration alone are insufficient for long-term recovery [8,20,21]. Thus, consideration of additional factors that may be associated with and contribute to this treatment profile is necessitated.

Clinical consensus accepts that psychological variables are affected by the acute stage of malnutrition. That is, starvation can result in psychological complications, including effects on mood and cognitive functioning [22,23]. Less is known, however, about the progression of psychological variables during the different stages of AN and their role in the maintenance of anorectic symptomology. Questions have repetitively been raised as to whether psychological symptoms should be regarded solely as complications of malnutrition, or whether they represent unique psychiatric features that need to be considered independently, and/or whether psychiatric comorbidity needs to be considered as normative. Despite increasing suggestions that core behavioral, attitudinal, and emotional disturbances [24,25] may persist after weight normalization and that core psychological difficulties do not automatically resolve following weight restoration in patients with AN [26-28], research remains limited.

A previous review addressing the aforementioned question considered both depressive and anxiety symptomology [29]. This review included 7 studies representing heterogeneous methodology and reporting largely contradictory findings, rendering a conclusive result impracticable [29]. There have been some theoretical suggestions that depressive symptomology in AN results in response to prolonged illness and symptom chronicity [30], whereas anxiety symptomology may increase vulnerability to eating disorder pathology [31]. Consistent with these suggestions, Hughes [32] in a comprehensive review of comorbid depression and anxiety in childhood and adolescent AN found that while anxiety symptoms tended to pre-date AN onset and persist following recovery, depression was likely to onset after AN and abate following recovery. As such, the current study chose to focus exclusively on anxiety symptomology in order to elucidate possible findings.

Focus on anxiety is further implicated by recent literature indicating high rates of psychiatric comorbidity between anxiety disorders and AN [33], marked symptomological overlap [28,34], evidence purporting the anxiolytic effects of AN behaviours [35], and more recent developments indicating a shared neurobiological profile [36-38]. Furthermore, there is some evidence to suggest that experiences of anxiety in populations of individuals with AN may be an indicator of poor treatment outcome [31]. In fact, Zerwas et al. [39] found that trait anxiety was a negative prognostic factor independently associated with AN recovery, and Thornton et al. [40] found that individuals with comorbid AN and Generalised Anxiety Disorder (GAD) diagnoses attained significantly lower BMI’s than individuals with AN only. Despite increasing evidence documenting the functional interplay between AN and anxiety, and suggestions pointing to the utility of understanding anxiety symptomology for efficacious treatment of AN [41], research remains limited.

Consequently, the current study sought to conduct a systematic review of research examining the relationship between anxiety symptomology and weight restoration in individuals with AN. Despite increasing evidence implicating the significance of anxiety symptomology in AN, the complex relationship of biological and psychological factors resulting in poor treatment outcomes appears to be poorly understood. This review aims to specifically clarify the expression of anxiety symptoms during treatment, and to determine the nature of the relationship between anxiety and weight normalization in AN patients.

## Method

### Search procedure and selection criteria

A comprehensive search of three electronic databases (PsycINFO, MEDLINE, and Web of Science) was conducted to identify relevant studies published before July 2014. Key search terms were (anorexia nervosa OR anorexia) AND (weight gain OR refeeding OR nutritional rehabilitation) AND (anxiety OR psychological stress OR distress OR fear OR psychological impact). This search identified 878 records, of which 150 were duplicates. Of the 728 records, a review of the titles and abstracts demonstrated that 642 did not meet inclusion criteria; these were omitted. A second author independently screened 10% of the titles and abstracts to control for selection bias, which demonstrated overall agreement in the selection of relevant articles chosen for review. The remaining 86 full-text articles were assessed for eligibility.

**Figure 1:**
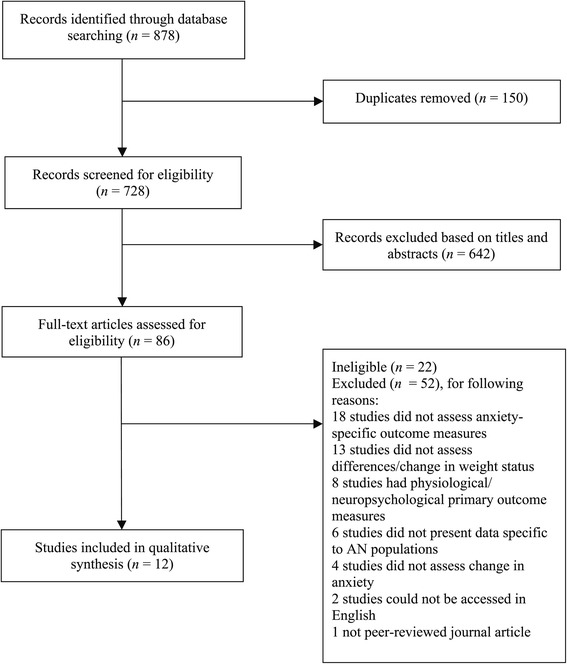
**Flow-diagram of selection process.**

To be eligible for inclusion, studies were required to have (a) examined a population with AN, (b) utilized a standardized measure of anxiety, (c) assessed change in weight status, (d) presented original data, and (e) been published in English in a peer-reviewed journal. To allow for comprehensive inclusion of studies from the available literature no restrictions were placed on (a) study design, (b) examination of the relationship between change in anxiety and change in weight status, and (c) criteria of age or gender of participants. Studies were excluded, however, if they did not explicitly examine an AN population (i.e., they included Eating Disorder Not Otherwise Specified or Bulimia Nervosa and did not report data specific to AN), or did not examine the primary outcomes of interest (e.g., measured depression only). Overall, 12 articles reached the standards stipulated by the inclusion and exclusion criteria. Figure 1 presents a flow diagram of the selection process.

### Data extraction

Data information collected included study design, demographic characteristics (age, gender, baseline BMI), diagnostic status (diagnostic criteria applied, AN subtype specification, medication use) and details of outcome measures (anxiety and weight measures, time point for measurements). Importantly, while studies documented a range of psychopathological variables (e.g., depression, motivation for change, obsessive compulsive disorder) and/or neuropsychological variables (e.g., executive functioning, processing speed) only data pertaining to anxiety was collected for the purpose of this systematic review. Further information pertaining to statistical analysis procedures and correlational outcome data specifically (i.e., relationship between anxiety and weight) was also extracted.

### Quality assessment

Study quality was assessed using a modified version of the original Quality Index by Downs and Black [42]. The Quality Index has been shown to be a valid and reliable tool for assessing methodological quality of epidemiologic and health research [42,43]. While the original tool was designed for systematic assessment of randomized and non-randomized studies of health care interventions, Ferro and Speechley [44], in their systematic review in the health science field, amended the tool to exclude items related specifically to interventions. As such, quality items assessing randomization, blinding, withdrawals and dropouts, and intervention integrity were excluded. As the current systematic review does not involve assessment of intervention studies, this modified Quality Index was deemed appropriate. The amended version (see Appendix 1) consists of 15 items scored dichotomously as 0 (No/Unable to determine) or 1 (Yes) resulting in three subscales; reporting (0 – 7), external validity (0 – 3), and internal validity (0 – 4), with a single item assessing study power. The maximum total score is 15, with higher scores indicated greater methodological quality.

## Results

Twelve articles were included in this systematic review. Studies were conducted in a number of different countries including France (*n* = 3), Italy (*n* = 2), United Kingdom (*n* = 2). United States of America (*n* = 2), Spain, Israel, and Norway. The majority of studies employed longitudinal designs (*n* = 6), with five studies utilizing a combination of cross-sectional and longitudinal data, and one study using cross-sectional data only [45]. The age of participants ranged from 14.4 to 29.86 years old. Tables 1 and 2 present the study characteristics and outcome data for those studies that included direct assessment of the relationship between anxiety and weight status (*n* = 5) and those that did not (*n* = 7).

**Table 1:** **Study characteristics for studies assessing the relationship between anxiety and weight**

**Citation**	**Country**	**Data**	**Time of evaluations/comparison groups**	**Refeeding protocol**	**Other treatment components**	**Sample** ***M age (SD)***	***N*** ^***c***^	**ED subtype (% of** ***N*** **)**	**Treatment duration** ***M*** **(** ***SD*** **)**	**Medication use** ^**d**^ **(% of** ***N*** **)**	**Anxiety measure (s)**	**BMI** ***M*** **(** ***SD*** **)**	**Anxiety change**	**Relationship BMI and anxiety**
Gauthier et al. [54]	France	Longitudinal	T1: Within 2 weeks of Ax	x	x	Inpatient 17.0 (3.25)	42 (2 males)	AN-R (45)	4.9 (4.37) months	Ax - 26.2^e^	HARS	14.1 (1.41)	NR*	*β* = − *0.121*
								AN – B/P (55)		Dx - 42.8^e^		17.5 (1.56)*		
			T2: Within 2 weeks of Dx			Controls	42					19.4 (2.24)		
Mattar et al. [56]	France	Longitudinal	T_Initial_: Within 1 week of Ax	Self-serviced and/or individually tailored supervised meal. NG if necessitated by illness severity^b^	x	Inpatient 6.38 (1.93)	24	AN-R (100)	3.2 (2.06) months	29.17^e^	HARS	13.84 (1.26)	NR*	*r* = 0.134^f^
			T_Final_: Within 1 week of Dx								STAI	17.79 (1.21)*		
			FU: 4 – 12 years after Dx^a^								- State		NR*	*r* = 0.248^f^
											- Trait		NR*	*r* = 0.112^f^
Morgan et al. [59]	UK	Longitudinal	1: Ax	Prescribed diet^b^	x	Inpatient 26.1 (3.52)	11	AN-R (54.5)	x	Unmedicated	HARS	16.1 (0.43)	*t* = −3.69*	*Mean slope = 0.10*
			2: Every 4 wks continuing for 8 weeks after MMPW (5 timepoints)					AN-BP (45.5)				20.4 (0.48)*		
Sala et al. [60]	France	Longitudinal	T1: One week observation	Individually tailored supervised diet^b^	Behavioural intervention	Inpatient 27.68 (6.54)	75	AN-R (55)	≈3 months	Unmedicated	STAI	14.90 (3.22)	*F* = 6.46^f^*	NR (ns)
												15.9 (2.95)		
			T2: 3-week regain phase									17.64 (2.63)		
												18.80 (2.22)*		
			T3: One week normalization					AN – B/P (30)				15.59 (1.97)		
												17.12 (1.98)		
			T4: One month stabilisation					BN (15)				19.19 (1.33)		
												19.52 (1.11)*		
Ricca et al. [52]	Italy	Longitudinal and cross sectional	T1: Ax	Unclear. Normal eating prescribed and weight range goal negotiated^b^	40-hour manual-based individual CBT	Outpatient 27.48 (±10.3)	53	NR	≈40 weeks	51.2%^e^	STAI	15.58 (1.69)	NR (ns)	NR (ns)
			T2: Dx								- State	17.28 (2.29)*		
			T3: 3 year FU								- Trait	17.23 (3.67)		
						Outpatient 29.86 (±8.93)	50	s-AN-WU (70%)		45.5%^e^		19.16 (1.86)		
												20.52 (3.70)*		
								s-AN-WA (30%)				19.54 (4.59)*		

Note. Ax: Admission; Dx: Discharge; FU: Follow-up; MMPW; Mean Matched Population Weight. NG: Nasogastric Tube Feeding. CBT: Cognitive Behavioural Therapy. AN-R: Anorexia Nervosa – Restrictive Subtype; AN-B/P: Anorexia Nervosa – Binge/Purging Subtype; BN: Bulimia Nervosa; s-AN: Subclinical AN; s-AN-WA: Subclinical Anorexia Nervosa without amenorrhea; s-AN-WU: Subclinical Anorexia Nervosa without weight criterion. HARS: Hamilton Anxiety Rating Scale [63]; STAI: State-Trait Anxiety Inventory [61]. BMI: Body Mass Index. X: Information not reported; NR: Statistic not reported; ns: Non-significant N/A: Not applicable.

*Significant at p < 0.05.

^a^Follow-up measurement timepoint not included in current review. ^b^Daily caloric intake not specified . ^c^All participants female unless otherwise indicated. ^d^Nil significant differences between patients on medication/not on medication unless otherwise indicated. ^e^Antidepressant medication only. ^f^Correlations are reported for BMI_Fiinal_ (i.e., BMI at discharge) Improvement and Anxiety Score Improvements at Discharge.

**Table 2:** **Study characteristics for studies that did not directly assess the relationship between anxiety and weight**

**Citation**	**Country**	**Data**	**Time of evaluations**	**Refeeding protocol**	**Other treatment components**	**Sample** ***M age (SD)***	***N*** ^***e***^	**ED subtype (% of** ***N*** **)**	**Treatment duration** ***M*** **(** ***SD*** **)**	**Medication use** ^**f**^ **(% of** ***N*** **)**	**Anxiety measure (s)**	**BMI** ***M*** **(** ***SD*** **)**	**Anxiety change**
Brambilla et al. [51]	Italy	Longitudinal & cross sectional	T1: Ax	Slow progressive introduction of micro and macronutrients^d^	CBT Psychopharmacology	Outpatient 22 (5)	22	AN-R (100)	x	Nortriptyline (50)	HARS	15.9 (1.9)	*F* = 25.3*
			T2: 1 month									18.3 (1.7)*	
			T3: 2 months									14.7 (1.5)	
			T4: 4 months of therapy							Fluoxetine (50)		16.3 (2.6)*	
Castro-Fornieles et al. [53]	Spain	Longitudinal	T1: Ax	Initiated on 1,500 kcal, progressive individually tailored increase to 2,500 kcal	Biological management & nutritional rehabilitation, behavioural program, group cognitive treatment & individual/group parent counselling	Inpatient 14.4 (1.7)	49 (1 male)	AN-R (75.5)	29.8 (17.6) days	24.5%^g^	STAI-Y	15.5 (1.4)	
			T2: Dx					AN-B/P (24.5)			- State	18.4 (0.8)*	*t* = 1.5
			T3: 9 months post Dx^a^								- Trait		*t =* 1.0
Dahlgren et al. [46]	Norway	Longitudinal	T1(Inpatient): *M* of 36 days post Ax	x	CRT	Inpatient & Outpatient	20	x	x	x	STAI	16.81 (1.63)	
			T1 (Outpatient: *M* of 13 months post Ax								- State	17.73 (1.39)*	*t* = 1.01
			T2 (All): Post CRT								- Trait		*t* = 1.54
Green et al. [47]	UK (?)	Longitudinal & cross sectional	TI (Inpatient): Ax	x	x	Inpatient 26	12	AN- R (100)	As previous	x	STAI	15.03 (0.71)	NR*
			T2: After 1 week								- State	15.02 (0.78)	
			T3: After 12 weeks of therapy				17				- Trait	16.53 (0.65)*	
			Controls: Over 12 weeks at same intervals as inpatients			Controls 21						21.82 (0.53)	
												21.82 (0.53)	
												21.85 (0.52)	
Lachish et al. [58]	Israel	Longitudinal & Cross sectional	T1: Within 7 days of Ax	x	SSRI and/or Atypical antipsychotics as required	Inpatient 15.9 (0.45)	24	AN-R (100)	4.23 (4.7) months	Ax – nil	STAI	15.5 (0.3)	NR (ns)
										Dx – 33.3%^gh^	State	19.5 (0.4)*	
			T2: Dx^b^								Trait	x	
			T3: 2 – 3 yr FU			Controls	19						
Perez et al. [48]	USA (?)	Longitudinal & cross sectional	T1: Ax	Caloric intake increased from <1200 kcal/day (T1) to ≥1800 kcal/day at T2*	x	Inpatient 15.5	16	x	As previous	56.25^i^	SCARED	17.3	NR (ns)
			T2: 14 (±2) weeks from Ax									18.32*	
						Controls 16.8	22			2^i^		20.7	
												21	
Pollice et al. [45]	USA (?)	Cross-sectional	1: Underweight	x	x	Inpatient 18 (±5)	22	AN-R (59%)	x	Unmedicated	HARS	72 (6)^j^	*F* = 19.29*
								AN-B/P (41%)			STAI	94 (3)^j^	
			2: Short-term weight restored								- State		*F* = 25.86*
			3: Long-term weight restored			Outpatient 24 (±4)	26				- Trait	98 (9)^j^	*F* = 22.31*
						Controls 23 (±4)	18	AN-R (50%)				104 (8)^j^	
			4: Controls^c^					AN-B/P (50%)					
								N/A					

Note. Ax: Admission; Dx: Discharge; FU: Follow-up. CRT: Cognitive Remediation Therapy; CBT: Cognitive Behaviour Therapy; SSRI: Selective Serotonin Reuptake Inhibitor. AN-R: Anorexia Nervosa – Restrictive Subtype; AN-B/P: Anorexia Nervosa – Binge/Purging Subtype. HARS: Hamilton Anxiety Rating Scale [63]; STAI-Y: STAI Child and Adolescent Version [62]; STAI: State-Trait Anxiety Inventory [61]; SCARED: Screen for Child Anxiety-Related Emotional Disorders [64]. BMI: Body Mass Index. X: Information not reported; NR: Statistic not reported; ns: Non-significant N/A: Not applicable.

*Significant at p < 0.05.

^a^Follow-up measurement not included in current review. ^b^Discharge defined by achievement and maintenance of desired weight for at least two consecutive weeks. ^c^Short-term weight restored measurements were obtained from the original sample of underweight patients within one month after achieving weight restoration. Long-term weight restored sample refers to individuals who maintained weight restoration for a period of 6 months to 10 years. ^d^Daily caloric intake not specified. ^e^All participants female unless otherwise indicated. ^f^Nil significant differences between patients on medication/not on medication unless otherwise indicated. ^g^No specification of type of pharmacological intervention provided. ^h^% relates to portion of participants retained at discharge (50% of original sample) on medication. ^i^Antidepressant or anxiolytic medication. ^j^Weight measurement reported as M (SD) % of average body weight recommended for their height according to the Metropolitan Life Insurance tables (Metropolitan Life Insurance Company [55]).

### Diagnostic criteria

Nine studies specified that participants were assessed according to diagnostic criteria for AN prior to study commencement; three studies did not specify psychiatric diagnostic criteria [46-48]. Perez et al. [48] specified admission criteria in regard to medical presentation (severe bradycardia, orthostatic hypotension, electrolyte abnormalities, inability to maintain weight and hypothermia) rather than applying psychological criteria. Of the nine studies utilizing specific diagnostic criteria, seven studies applied DSM-IV or DSM-IV-TR criteria [49], one study [45] applied DSM-III-R criteria [50], and one study utilized both DSM-III-R and DSM-IV criteria [51]. These applications represent appropriate use of diagnostic manuals that were available at the time of study publication.

In addition to diagnostic criteria, subtype specifications were documented in ten studies. Percentage of total sample was documented for each subtype, such that the percentage of participants with AN Restrictive Subtype ranged from 45 to 100%, while participants with AN Binge/Purging Subtype were less represented with percentages ranging from 24.5 – 55%. Additional subtypes were stipulated in one study [52], which considered two types of atypical/subclinical anorexia nervosa presentations where all diagnostic criteria were met except underweight status or amenorrhea. Notably, these subtype specifications may no longer be applicable as a result of the introduction of DSM-5 [2] that allows for greater variability in weight status at presentation accounted for by a severity specifier.

### Participant sample

Eight studies’ clinical samples were inpatient populations only, two studies assessed an outpatient population only [51,52], and two studies [45,46] assessed participants from both inpatient and outpatient units. The number of participants varied across the studies with baseline samples ranging from 11 to103 (*M* = 47). Half the studies assessed adult populations, while half the studies assessed adolescent populations. Importantly, only two studies identified the inclusion of male participants [53,54] with those male participants representing only 2% and 5% of the total sample, respectively. Five of the included studies utilized a non-eating disordered control group.

### Weight measurements

All studies collected objective measurements of weight and height, with 11 studies reporting Body Mass Indices [*BMI* = weight (kg)/height (m^2^)] and one study reporting percentage of ideal body weight [45]. Baseline weight measurements for clinical samples were consistent with DSM-IV [49] diagnostic indications of anorexia nervosa (i.e., BMI <17.5) with average baseline BMI ranging from 13.84 to 17.37 (*M* = 15.61). Pollice et al. [45] reported that those participants in the underweight anorexia nervosa group, consistent with diagnostic criterion, were on average at 72% of the average recommended body weight for their height according to the Metropolitan Life Insurance Tables [55]. Some studies provided detailed procedures for collection of weight measurements, for example Mattar et al. [56] who indicated that adolescents were weighed wearing light clothing and were subsequently categorized according to Cole’s index of thinness [57], while the majority of studies simply stated that measurements were taken at session commencement or did not report specific measurement procedures.

Of the five studies including control populations, three studies [47,48,54] reported baseline BMI status, which fell within healthy parameters ranging from 19.4 to 21.82 (*M* = 20.64). Pollice et al. [45] documented that on average control participants were at 104% of the average recommended body weight for their height according to the Metropolitan Life Insurance Tables [55]. Lachish et al. [58] defined inclusion criteria for control women on the basis of weight status falling within 85 – 115% of ideal body weight according to the Metropolitan Life Insurance [55] criteria but did not report specific weight data for the control group. All eleven studies reporting on longitudinal data indicated a significant increase in BMI during the course of treatment, with discharge BMI ranging from 16.53 [48] to 20.4 [59].

### Re-feeding protocol and additional treatment components

Seven studies provided a description of refeeding protocol applied for weight restoration. Of those seven, only two studies [44,47] provided a description in terms of daily caloric intake. Castro-Fornieles et al. [53] initiated participants on meal plans of 1,500 kcal/day that increased to 2500 kcal/day by the end of their admission. Perez et al. [48] described that daily caloric intake increased from less than 1200 kcal/day at admission to greater than 1800 kcal/day at discharge; however, these authors did not provide specification of procedure for increased intake. The remaining five studies provided brief and often vague descriptions of individualized meal plans designed for nutritional rehabilitation.

### Treatment duration and measurement time points

Treatment duration varied according to the model of intervention and adopted definitions of recovery. Specific discharge criteria were largely not reported; however, all studies utilizing an inpatient population indicated a predominant goal of weight restoration. Castro-Fornieles et al. [53] stipulated that both weight recovery and normalization of eating patterns were required prior to discharge, while Morgan et al. [59] indicated that individuals must reach a mean matched population weight (MMPW) prior to discharge. Four studies reported specific data for average treatment duration, with length of hospitalization ranging from 29.8 days [53] to 4.9 months [54]. Treatment duration for four additional studies could be discerned from descriptions of treatment components. Sala et al. [60] reported a prescriptive treatment length of 3 months based on four phases of treatment. Ricca et al. [52] reported a 40-week treatment protocol. While Green et al. [47] and Perez et al. [48] indicated total specific measurement periods, 12 weeks and 14 weeks (±2) respectively; it was unclear whether this represented the full treatment duration. Two studies [46,59] did not report on treatment duration.

In terms of measurement time points, all eight studies utilizing inpatient populations only, reported outcomes at least in relation to admission and discharge. There was variability, however, in terms of allowed lapsed time in relation to these treatment qualifiers. For instance, Morgan et al. [59] reported a mean lag time of 18.5 days (*SD* = 3.89) between admission and recruitment into the study as a result of hospital protocol designed to prevent research impediment on assessment process. Mattar et al. [56] and Lachish et al. [58] collected data within one week of admission, whilst Gauthier et al. [54] allowed for measurement within two weeks of admission. Dahlgren et al. [46] reported a mean lapse between treatment admission and baseline measurement of 36 days for their inpatient population and 13 months for their outpatient population.

In addition to baseline and discharge measures, four studies included measurements at multiple time-points. Brambilla et al. [51] and Morgan et al. [59] reported monthly measurements throughout treatment, while Green et al. [47] included an additional measurement time-point one week after admission. Sala et al. [60] reported measurements at each defined phase of treatment. Four studies reported follow-up periods that ranged from 9 months post-discharge [53] to 12 years [56], however, for the purposes of the current systematic review these assessments were not included. Pollice et al. [45] used a cross-sectional study design and as such only have one measurement time-point.

### Medication

Ten studies accounted for the use of psychopharmacology, while two studies [46,47] did not provide any information. Of those studies reporting psychopharmacological data, six studies reported on antidepressant medication with use at admission ranging from 0 [58] to 51.2% [52]. Perez et al. [48] accounted for use of antidepressant and anxiolytic medication with 56.25% of their inpatient population reportedly on medication. Ricca et al. [52] excluded participants if they were currently using any other psychoactive medication, other than of anti-depressant medication. Two studies reported percentage of participants on antidepressant medication at admission and discharge [54,58], with both studies reporting a relative increase in medication use during hospitalization. Importantly, with the exception of one study [51], which was designed to assess the relative impact of two antidepressant medications (nortriptyline vs. fluoxetine), where medication status was reported use of antidepressant medication was not significantly related to outcome. Three studies [45,59,60] stipulated that participants did not receive any psychotropic medication prior to or during the study period.

### Anxiety measures

Anxiety symptomatology was measured using a number of different validated self-report questionnaires. The majority of studies only included one measure of anxiety; however, two studies [45,56] used two measures of anxiety. Seven studies used the State Trait Anxiety Index (STAI) [61], which is a widely used self-report instrument that yields separate scores for state and trait anxiety experiences. One study [53] utilized a version of the STAI that had been adapted for child and adolescent populations (STAI-C) [62]. The well-validated Hamilton Rating Scale for Anxiety (HARS) [63] was used by five studies to measure behavioral and somatic symptoms associated with anxiety in the previous week. Perez et al. [48] used the Screen for Child Anxiety-Related Emotional Disorders (SCARED) [64], which is a self-report instrument designed to screen for the presence of anxiety in child and adolescent populations aged 9 – 18 years.

### Change in anxiety symptomology

Seven studies reported a significant change in anxiety symptomology, such that anxiety symptomology seemed to recede during the course of treatment. Three of these studies did not include statistical figures related to this finding. Pollice et al. [45] in the only cross-sectional study found that, whilst anxiety ratings were highest in underweight anorexia nervosa patients, short-term and long-term recovered patients with AN demonstrated somewhat reduced levels of anxiety; however, these were still elevated when compared to healthy controls. In contrast to these significant findings, the remaining five studies found no evidence of a significant change in anxiety symptomology during the course of weight restoration.

### Relationship between weight status and anxiety symptomology

All five studies assessing the relationship between weight status and anxiety symptomology failed to find a significant relationship. That is, despite significant changes in weight status and significant changes in anxiety symptomology, these two factors were not associated.

### Evaluation of study quality

Quality ratings for individual studies are detailed in Table 3. Quality scores ranged from 7 to 12, with an average quality rating of 11.42 out of a possible 15. In terms of subscale indices, scores for reporting quality ranged from 5 to 7 (*M* = 6.41/7), for external validity ranged from 0 to 3 (*M* = 1.67/3) and for internal validity ranged from 2 to 3 (*M* = 2.25/4). Only one study [48] provided a power calculation. Higher scores represent greater methodological rigour, thus, these results indicate that studies were scoring better for the reporting and internal validity subscales than for the external validity subscale.

**Table 3:** **Quality index of included studies (Ferro and Speechley** [44]**, amended from Downs and Black** [42]**)**

	**Hypothesis clearly described**	**Main outcomes clearly described**	**Characteristics of patients described**	**Main findings clearly described**	**Estimates of random variability**	**Actual probability values used**	**Response rate clearly described**	**Patients-represent population**	**Patients prepared-represent population**	**Staff, place and facilitates**	**Data dredging**	**Statistical test procedures**	**Outcome measures valid/reliable**	**Adjustment for confounding**	**Sample size or power calculation**	**Total**
Brambilla et al. [50]	1	1	1	1	1	1	0	0	1	1	0	1	1	0	0	10
Castro-Fornieles et al. [52]	1	1	1	1	1	1	1	1	1	1	0	1	1	0	0	12
Dahlgren et al. [45]	1	1	1	1	1	1	1	0	0	1	0	1	1	0	0	10
Gauthier et al. [53]	1	1	1	1	1	0	1	1	0	1	0	1	1	1	0	11
Green et al. [46]	1	1	1	1	1	1	0	0	0	1	0	1	1	0	0	9
Lachish et al. [57]	1	1	1	1	1	1	1	0	0	1	0	1	1	1	0	11
Mattar et al. [55]	1	1	1	1	1	1	1	1	0	1	0	1	1	1	0	12
Morgan et al. [58]	1	1	1	1	1	1	1	1	1	1	0	1	1	0	0	12
Perez et al. [47]	1	1	1	1	1	1	0	0	0	1	0	1	1	0	1	10
Pollice et al. [44]	1	1	1	1	1	0	0	0	0	0	0	1	1	0	0	7
Ricca et al. [51]	1	1	1	1	1	1	1	1	1	1	0	1	1	0	0	12
Sala et al. [59]	1	1	1	1	1	0	1	0	0	1	0	1	1	0	0	9

## Discussion

This systematic review aimed to critically evaluate existing literature regarding anxiety symptomology in relation to weight restoration in populations with AN. Despite increasing evidence implicating the need to understand anxiety within AN populations [41] and the way in which anxiety may contribute to the maintenance of symptoms in patients with AN [40], research remains limited. Twelve studies, published between 1995 and 2014, fulfilled the criteria for review. Results regarding change in anxiety symptoms during hospitalization for weight restoration were inconsistent across studies, with some studies reporting a decrease in anxiety and a similar portion reporting null findings. Evidence explicitly examining the relationship between weight status and anxiety symptomology was relatively sparse; overall it indicated a non-significant association.

### Summary of findings

Six studies reported a significant change in anxiety, such that anxiety symptoms decreased over the course of treatment. Interestingly, only two of these studies [51,60] indicated that they had employed some form of psychological intervention, seemingly suggesting that anxiety symptoms recede during treatment aimed at weight restoration despite not being directly targeted. These findings are consistent with neurobiological models that suggest that psychopathology can be confounded by malnutrition associated with starvation in populations with AN [22]. Notably, the majority of those studies reporting a reduction in anxiety indicated that despite the significant decrease, anxiety symptomology did not resolve entirely, remaining at levels above those expected for non-clinical populations. Inconsistent to the abovementioned studies, an equivalent number of studies included in this review did not find a significant change in anxiety symptoms over the course of hospitalization. Taken together, these finding seems to support suggestions for the persistence of symptomology despite weight normalization [24,25]. Alternatively, the apparent lack of reduction in anxiety may in fact relate to a putative increase in anxiety associated with weight gain in a population diagnostically defined as having a fear of gaining weight [2]. There is clearly a need for further research to better understand this phenomenon.

All five studies assessing the relationship between weight status and anxiety symptomology did not find a significant association between these two variables. Importantly, all of these studies reported improvement in BMI and, with the exception of one study [52], an improvement in anxiety symptomology. While BMI change did not always reflect an achievement of normative weight levels, leaving the possibility for further improvement and significant findings once weight normalization is achieved, it seems unlikely that these null findings are attributable to non-meaningful weight change. On the basis of their null findings, Sala et al. [60] concluded that extremely elevated baseline anxiety pathology can reduce to moderate levels but may otherwise persist in AN populations. Mattar et al. [56] concluded that it was unclear which components of anxiety were related specifically to malnutrition and which components may reflect concomitant anxiety for the individual, independent of malnutrition. Gauthier et al. [54] extended this explanation by suggesting that anxiety symptoms linked to malnutrition recede but that symptoms independent from the nutritional state may persist.

The aforementioned suggestions are consistent with research indicating that weight restoration does not automatically result in the resolution of anxiety and core psychological difficulties in patients with AN [26-28]. Given that anxiety may be a negative prognostic factor for recovery [31,39] and that long-term treatment outcomes remain poor [5], future research explicitly examining the temporal relationship of anxiety and the treatment of individuals with AN may delineate these propositions and assist in developing an understanding of psychological factors that contribute to the pathogenesis and maintenance of the disorder. The consideration of comorbid anxiety disorders and resultant anxiety symptomology as independent from eating disorder psychopathology may additionally extend this understanding. Notably, only two of the included studies [52,58] explicitly measured comorbid anxiety disorders. Despite the evident utility of research in this area, only a handful of studies have been conducted.

Given the scarcity of research within the area, inclusion criteria concerning methodological design were kept broad for this systematic review. While this allows for a wide-ranging assessment of all available literature, it is also a limitation of the current study. The heterogeneity of included studies may explain the lack of consistent findings in regards to change in anxiety symptomology. Treatment periods were highly variable, ranging from 29.8 days to 4.9 months. While this reflects observations across clinical settings [65], it may limit meaningful comparisons. Additionally, treatment approaches varied markedly. Some studies utilized additional behavioural and psychological interventions, whilst others did not indicate any specialist intervention beyond nutritional rehabilitation. Nevertheless, there is no empirical evidence implicating the use of any one-treatment setting (i.e., inpatient, outpatient, or day program) over the other [66,67]. Furthermore, sample characteristics (e.g., participant age, treatment setting) varied across studies. No meaningful categorization of these variables seemed applicable in the current study; however, future research may want to further classify these factors.

A number of methodological strengths of the available literature should be accounted for. These include the use of appropriate criteria and health-professional interviews to enable a clinical diagnosis of AN, largely valid and reliable methods of data collection and recruitment, objective measurements of BMI, and the use of widely used, validated and reliable measures to assess anxiety. Furthermore, in general, quality ratings across all studies were moderate. Mean scores for the internal validity subscale of the Quality Index, in particular, suggest that studies were adequate in producing internally valid results. Mean scores for the external validity subscale of the Quality Index, however, imply the need for caution when generalizing results. Patients across studies were recruited predominantly from secondary and tertiary referral services (i.e., inpatient and outpatient services). Whilst some studies included patients with sub-clinical symptomology, findings cannot be generalized to non-treatment seeking populations.

Despite these relative strengths, there are a number of limitations evident in the reviewed literature. Poor treatment outcome in AN is invariably associated with illness chronicity and increased duration of symptology prior to treatment intervention [5]. While the majority of studies reported on these variables at baseline and/or included exclusion criteria in an attempt to account for these variables, they were largely unaccounted for in statistical analyses. Furthermore, while the majority of the studies accounted for the concurrent use of antidepressant medication, other psychotropic medications were either unaccounted for or participants were excluded on the basis of their use. Evidence around the efficacy of these psychopharmacological interventions is sparse and largely inconclusive [68,69]. Nevertheless, atypical anti-psychotic medication is increasingly used in the routine treatment of AN patients [70] as a result of models purporting the utility of the desirable side effect of weight gain as well as their presumed effectiveness in targeting anxiety symptomology. Thus, it seems pertinent to rigorously account for these psychopharmacological interventions in studies designed to assess weight restoration and anxiety symptomology in AN populations. Notably, some of the studies included in the current review measured anxiety as a secondary outcome and as such did not formulate study aims around the question prescribed in the current systematic review.

Limited information about specific nutritional treatment and caloric intake was provided by included studies. At present, there are no clear recommended protocols for ideal initial doses of nutrition [9,71]. However, emerging research suggests that traditional refeeding programs, designed to prevent the onset of refeeding syndrome, may be too conservative [9]. As such, more aggressive approaches to weight restoration (in the context of intensive metabolic monitoring) are emerging due to their propensity to promote more efficient weight gain, reduction in length of hospital stays and effective minimization of the financial burden of inpatient care, without compromising physiological well-being [71-73]. While there is some suggestion that the rate of weight restoration during inpatient treatment can be a significant predictor of short-term clinical outcomes [74], research regarding the psychological tolerability of rapid-refeeding protocols is deficient. Studies assessing the psychological impact of weight restoration should invariably provide a detailed description and analysis of the applied refeeding protocols to provide clarification.

## Conclusions

Taken together, the results of the current review highlight marked inconsistencies in evidence regarding change in anxiety symptoms during treatment. These findings may be limited by numerous methodological shortcomings, including inconsistencies in regards to interventions and length of treatment, poor temporal assessment of weight gain, as well as lack of control over significant confounding variables (e.g., concurrent psychopharmacological intervention, history and/or course of illness, comorbid psychiatric diagnoses). The methodological shortcomings render it almost impossible to draw meaningful conclusions. Developing an understanding of additional factors that contribute to poor weight normalization and conceptualizations of factors that contribute independently to the maintenance of anorectic symptoms is necessitated. Additional research into alternate psychological factors such as co-morbid depression [75] or motivational factors [76] may be useful.

In regards to the relationship between anxiety symptoms and weight restoration, the results of the current review do not support a significant association. In line with this finding, evidence points towards the insufficiency of solely targeting weight restoration for long-term recovery. While research affirms the importance and obligatory targeting of weight restoration in the recovery of AN [25], there are increasing suggestions that marked gaps in the relative pace of physical and psychological recovery [77] may hinder overall progress and attainment of desirable clinical outcomes. Furthermore, Strober and Johnson [78] suggested that there is no evidence – clinical or empirical – to indicate that normal weight is a necessary prerequisite for initiating meaningful psychotherapeutic interventions, which may facilitate weight change. There is a current lack of empirical data, thus rigorous research that directly assesses these propositions is urgently needed.

## Appendix 1

Quality Index by (Ferro and Speechley [44]; amended from Downs and Black [42]).

*Reporting*

1. Is the hypothesis/objective of the study clearly described?

2. Are the main outcomes to be measured clearly described in the Introduction/Method section?

3. Are the characteristics of the patients included in the study clearly described?

4. Are the main findings of the study clearly described?

5. Does the study provide estimates of the random variability in the data for the main outcome?

6. Have actual probability values been reported for the main outcomes except where the probability value is less than 0.001?

7. Is the response rate clearly described?

*External Validity*

8. Were the patients asked to participate in the study representative of the entire population from which they were recruited?

9. Were patients who were prepared to participate representative of the entire population from which they were recruited?

10. Were the staff, places and facilities where the patients were studied, representative of the treatment the majority of patients receive?

*Internal Validity (Bias and confounding)*

11. If any of the results of the study were based on “data dredging”, was this made clear?

12. Were the statistical tests used to assess the main outcomes appropriate?

13. Were the main outcome measures used valid and reliable?

14. Was there adequate adjustment for confounding in the analyses from which the main results were drawn?

*Power*

15. Did the study provide a sample size or power calculation to detect important effects where the probability value for a difference being due to change is less than 0.05?
